# Identification in Chinese patients with *GLIALCAM* mutations of megalencephalic leukoencephalopathy with subcortical cysts and brain pathological study on *Glialcam* knock-in mouse models

**DOI:** 10.1007/s12519-019-00284-w

**Published:** 2019-08-01

**Authors:** Zhen Shi, Hui-Fang Yan, Bin-Bin Cao, Mang-Mang Guo, Han Xie, Kai Gao, Jiang-Xi Xiao, Yan-Ling Yang, Hui Xiong, Qiang Gu, Ming Li, Ye Wu, Yu-Wu Jiang, Jing-Min Wang

**Affiliations:** 1 Department of Pediatrics, Peking University First Hospital, No. 1 Xi’an Men Street, West District, Beijing, 100034 China; 2 Beijing Key Laboratory of Molecular Diagnosis and Study on Pediatric Genetic Diseases, Peking University First Hospital, Beijing, 100034 China; 3 Guangzhou Women and Children’s Medical Center, Guangzhou, 510623 China; 4 Department of Pediatrics, Beijing Tian Tan Hospital, Capital Medical University, Beijing, 100050 China; 5 Department of Radiology, Peking University First Hospital, Beijing, 100034 China; 6 Key Laboratory for Neuroscience, Ministry of Education/National Health Commission of the People’s Republic of China,, Peking University, Beijing, 100034 China

**Keywords:** *GLIALCAM*, Knock-in mouse model, Macrocephaly, Megalencephalic leukoencephalopathy with subcortical cysts, Vacuolization

## Abstract

**Background:**

Megalencephalic leukoencephalopathy with subcortical cysts (MLC) is a rare neurological degenerative disorder caused by the mutations of *MLC1* or *GLIALCAM* with autosomal recessive or autosomal dominant inheritance and a different prognosis, characterized by macrocephaly, delayed motor and cognitive development, and bilateral abnormal signals in cerebral white matter (WM) with or without cysts on magnetic resonance imaging (MRI). This study aimed to reveal the clinical and genetic features of MLC patients with *GLIALCAM* mutations and to explore the brain pathological characteristics and prognosis of mouse models with different modes of inheritance.

**Methods:**

Clinical information and peripheral venous blood were collected from six families. Genetic analysis was performed by Sanger sequencing of *GLIALCAM*. *Glialcam*^Arg92Trp/+^ and *Glialcam*^Lys68Met/Thr132Asn^ mouse models were generated based on mutations from patients (c.274C>T(p.Arg92Trp) (c.203A>T(p.Lys68Met), and c.395C>A (p.Thr132Asn))). Brain pathologies of the mouse models at different time points were analyzed.

**Results:**

Six patients were clinically diagnosed with MLC. Of the six patients, five (Pt1–Pt5) presented with a heterozygous mutation in *GLIALCAM* (c.274C>T(p.Arg92Trp) or c.275G>C(p.Arg92Pro)) and were diagnosed with MLC2B; the remaining patient (Pt6) with two compound heterozygous mutations in *GLIALCAM* (c.203A>T (p.Lys68Met) and c.395C>A (p.Thr132Asn)) was diagnosed with MLC2A. The mutation c.275C>G (p.Arg92Pro) has not been reported before. Clinical manifestations of the patient with MLC2A (Pt6) progressed with regression, whereas the course of the five MLC2B patients remained stable or improved. The *Glialcam*^Arg92Trp/+^ and *Glialcam*^Lys68Met/ Thr132Asn^ mouse models showed vacuolization in the anterior commissural WM at 1 month of age and vacuolization in the cerebellar WM at 3 and 6 months, respectively. At 9 months, the vacuolization of the *Glialcam*^Lys68Met/ Thr132Asn^ mouse model was heavier than that of the *Glialcam*^Arg92Trp/+^ mouse model. Decreased expression of Glialcam in *Glialcam*^Arg92Trp/+^ and *Glialcam*^Lys68Met/ Thr132Asn^ mice may contribute to the vacuolization.

**Conclusions:**

Clinical and genetic characterization of patients with MLC and *GLIALCAM* mutations revealed a novel mutation, expanding the spectrum of *GLIALCAM* mutations. The first *Glialcam* mouse model with autosomal recessive inheritance and a new *Glialcam* mouse model with autosomal dominant inheritance were generated. The two mouse models with different modes of inheritance showed different degrees of brain pathological features, which were consistent with the patients’ phenotype and further confirmed the pathogenicity of the corresponding mutations.

**Electronic supplementary material:**

The online version of this article (10.1007/s12519-019-00284-w) contains supplementary material, which is available to authorized users.

## Introduction

Megalencephalic leukoencephalopathy with subcortical cysts (MLC, OMIM 604004) is an infantile-onset autosomal recessive or dominant neurodegenerative disease characterized by cerebral white matter (WM) edema and cyst formation [1, 2]. The classic phenotype of MLC is macrocephaly within the first year of life, moderate to severe motor development delay, normal or mild mental development delay, motor deterioration with ataxia, seizures, spasticity in subsequent years, and extrapyramidal outcomes [1, 2]. Magnetic resonance imaging (MRI) displays bilateral symmetric diffuse abnormal signals in WM with edema, with or without subcortical cysts in the anterior-temporal and fronto-parietal lobes; the internal capsule, cerebellum, and brainstem are occasionally involved [1, 3]. The lifespan ranges from toddler age to seventies [4–8]. *MLC1* (OMIM 605908) was the first gene found to be mutated in MLC in 2001 [9]. Mutations in *MLC1* lead to classic MLC, which is diagnosed as MLC1 (OMIM 604004) and account for 75% of patients with MLC. The clinical and genetic characteristics and natural history of MLC1 have been completely studied [10–15]. The second gene *GLIALCAM* was found mutated in 2011 [4]. The phenotype associated with *GLIALCAM* is named as MLC2. MLC2 can be further divided into two types: MLC2A with autosomal recessive and MLC2B with autosomal dominant inheritance, and MLC2 patients accounted for a total of 20% of MLC patients. MLC2A and MLC2B present with different clinical symptoms and prognosis [4]. So far, only 89 patients with MLC and *GLIALCAM* mutations have been reported in five papers [4, 8, 11, 12, 16]. The brain tissues of patients with MLC are rare for *MLC1* mutations and unobtainable for *GLIALCAM* mutations. Therefore, MLC mouse models are important for studying the brain pathophysiology of this disorder. In the present study, we aimed to reveal the clinical characteristics, including those in long-term follow-up studies, genetic features, and phenotype–genotype relationship in MLC patients with *GLIALCAM* mutations and generate *Glialcam* mouse models with autosomal recessive and autosomal dominant inheritance to clarify the disease course and understand the brain pathological features of mouse models with different modes of inheritance.

## Methods

### Clinical analysis

Patients were enrolled from the Pediatric Neurology Department, Peking University First Hospital from 2007 to 2017 in accordance with the following criteria: (1) diagnosed with MLC clinically; (2) no pathogenetic mutation in *MLC1* was detected by sanger sequencing. Clinical information, such as visiting age, sex, onset age, presenting symptoms, and MRI results, was collected. The study was approved by the Ethics Committee of Peking University First Hospital, and written informed consents was obtained from the patients’ legal guardians.

Follow-up study data, including head circumference (HC), motor and mental development or retrogress, cranial MRI, head trauma histories, and other related symptoms, were collected by telephone follow-up survey. Motor function was assessed using the Chinese version of Gross Motor Function Classification System (GMFCS) for children with cerebral palsy [12]. The classification from GI to GV was represented as slightly to severe limited motor ability based on the patients’ age and motor function. Cognitive function was evaluated by word expression. The classification from C0 to C3 represented different levels of cognitive capacity from high to low according to their scores.

### Genetic analysis

Peripheral venous blood samples were collected from six patients and their family members. DNA extraction, amplification, and sequencing of *GLIALCAM* were performed as previously reported [11, 12]. The sequence variants were screened and analyzed using a flexible function converged online tool for detailed annotation Variant Effect Predictor (VEP, https://asia.ensembl.org/Homo_sapiens/Tools/VEP) [17]. Finally, a comprehensive prediction was analyzed in accordance with the 2013 ACMG standards and guidelines [18].

### Animals 

Mutations were derived from Chinese MLC patients with *GLIALCAM* mutations c.274C>T, p.Arg92Trp, c.203A>T(p.Lys68Met), and c.395C>A (p.Thr132Asn). Adult *Glialcam*^Arg92Trp/+^, *Glialcam*^Lys68Met/+^, and *Glialcam*^Thr132Asn/+^ point mutation mice were obtained from the Beijing Vitalstar Biotechnology Co., Ltd. using a CRISPR/Cas9-based knock-in strategy in C57Bl/6N mice (Fig. S2A). Then, we crossbred heterozygous mutant mice between *Glialcam*^Lys68Met/+^ and *Glialcam*^Thr132Asn/+^ mice to obtain the compound heterozygous mouse *Glialcam*^Lys68Met/Thr132Asn^. The study was approved by the Animals Ethical Committee of Peking University First Hospital, and animal housekeeping and experiments were in compliance with the guidelines of the committee. All mice were housed in an animal care facility (SPF) with a 12-h light–dark schedule and with freely available food and water. The DNA of the tail was extracted using the KAPA Express Extract kit, and genotype identification of these mice was performed by PCR and Sanger sequencing using the following primers: forward primer 5ʹ-AGGGCTTGAAG TTGGAAATGGGCTA-3ʹ; reverse primer 5′-AATTACCACTCAGCCTTTGCGTTGC-3ʹ. Table S1 records the sex and generations of mice used for the experiment.

### HE staining and vacuole quantification

Mice were anesthetized by 0.5% pentobarbital sodium at the dosage of 50 mg/kg, perfused by 0.9% saline and 4% paraformaldehyde. Brains were then perfusion-fixed with 4% paraformaldehyde and embedded in paraffin. Tissue slices were stained with hematoxylin and eosin (HE). Slice series from brain sagittal sections stained with HE were obtained through scanning by NanoZoomer RS 2.0.0 (HAMAMATSU, Japan) at 40× objective. Three slices of each animal brain were obtained, and three different animals of each genotype at four different time points were analyzed. Blind quantification of the vacuoles in the cerebellar and anterior commissural WM was performed to the genotype using ImageJ.

### Fluorescence immunohistochemistry

Fluorescence immunohistochemistry staining of Glialcam and Gfap were used to check the expression and localization of Glialcam among different group. For each genotype, three brain sections each of three different mice were analyzed by the Olympus confocal microscope and FV3000 system (Tokyo, Japan).

### Statistical analyses

WM vacuolization was analyzed using one-way ANOVA using SPSS version 19 (Armonk, USA) and GraphPad Prism 5 (San Diego, Canada). A *P* value < 0.05 was considered as statistically significant.

## Results

### Clinical and genetic findings of patients with MLC and GLIALCAM mutations

#### Clinical and follow-up findings

Six patients (Pt1–Pt6) enrolled in this study were clinically diagnosed with MLC (Table 1). One patient (Pt6) has been reported twice and three patients (Pt1–Pt3) have been reported once in two follow-up studies [11, 12]. The two patients (Pt5 and Pt6) were new-coming, and another three follow-up studies were performed for all six patients. The clinical characteristics of four patients were macrocephaly, earlier normal motor development, and classic MRI. Three patients were diagnosed as MLC2B and one as MLC2A. The patient with MLC2A showed motor retrogress, while one patient with MLC2B had an improvement in the MRI. For all six patients, the onset age ranged from birth to 7 months, with a median of 4 months. Macrocephaly was noted in five patients (Pt1–Pt4 and Pt6) with HC above the 97th percentile. Pt5 presented with HC > 1 SD history. In all six patients, the development of earlier motor and a rapid HC growth milestones, such as head control, rolling over, and sitting alone, were all normal (Table 2). However, the patients spent more time from walking unstably to walking stably. Motor developmental delay was observed in Pt4 and Pt6. Cognitive development was normal in all patients, except in Pt4, at the first visit (Table 3). Autism and language development delay were found in Pt4. Epilepsy occurred in Pt5. Transient macrocephaly was found in the mothers of Pt1 and Pt4 before 1 year old. Hypotonia was found in Pt2 and Pt3. Motor retrogress after head trauma was found in Pt6. Bilateral diffuse cerebral abnormal signals in swollen WM with or without cysts were found in the MRIs of all patients, expect Pt5, at the first visit. In Pt5, only subcortical WM was involved in the MRI. Posterior limb of the internal capsule was involved in Pt1 and Pt3.

**Table 1:** The Clinical characteristic of 6 patients with MLC at first visit

Pt	Gender	Age^visit^	Age^onset^	Symptom^Onset^	HC (cm)	EPS	FH	Seizures	Cranial MRI /CT					
									Age	Cerebral WMA	Cerebral WMS	PLIC	ALIC	Cysts
1	M	1 y 5 mon	0 mon	Mc	53 (> 2 SD)	−	+	−	7 mon	+	+	+	−	+
									7 y 9 mon	−	−	−	−	−
2	M	9 mon	5 mon	Mc	50 (> 2 SD)	+	−	−	8 mon	+	+	+	−	+
3	M	10 mon	4 mon	Mc	50 (> 2 SD)	+	−	−	8 mon	+	+	−	−	+
4	M	1 y 2 mon	7 mon	Mc	52 (> 2 SD)	−	+	−	8.5 mon	+	+	−	−	+
5	M	2 y 10 mon	2 y 10 mon	Seizures	51 (> 1 SD)	−	−	+	2 y 4 mon	+	+	−	−	−
6	F	5 y	4 mon	Mc	55 (> 2 SD)	−	−	−	2 y	+	+	/	/	+

*F* female, *M* male, *Age*^*visit*^ at first visit age, *Age*^*onset*^ onset age, *Symptom*^*Onset*^ onset symptom, *HC* head circumference(cm), *EPS* extrapyramidal signs, *FH* family history, *WMA* white matter abnormality, *WMS* white matter swelling, *PLIC* posterior limb of internal capsule, *ALIC* anterior limb of internal capsule, / not done, + positive, − negative

**Table 2:** The motor milestones, GMFCS and expression evaluation results of 6 patients at first visit and five times follow-up

Pt	Sex	HCt	RO	IS	IW	WS	WE	At first visit			1st follow-up			2nd follow-up			3rd follow-up			4th follow-up			5th follow-up		
								Age	G	C	Age	G	C	Age	G	C	Age	G	C	Age	G	C	Age	G	C
1	M	7 mon	8 mon	8 mon	1 y 1 mon	2 y	13 mon	1 y 5 mon	I	0	4 y	I	0	5 y 5 mon	I	0	7 y 6 mon	N	0	9 y 3 mon	N	0	10 y 9 mon	N	0
2	M	3 mon	3 mon	7 mon	NA	NA	NA	9 mon	I	0	NA			NA			NA			NA			NA		
3	M	4 mon	5 mon	7 mon	1 y 9 mon	2 y	1 y 6 mon	10 mon	I	0	1 y	I	0	2 y 3 mon	I	0	4 y 3 mon	N	0	6 y 1 mon	N	1	NA		
4	M	5 mon	7 mon	9 mon	2 y 8 mon	3 y 6 mon	NA	1 y 2 mon	I	1	NA			NA			NA			2 y 3 mon	I	2	3 y 8 mon	I	3
5	M	3 mon	6 mon	7 mon	1 y 4 mon	1 y 6 mon	1 y	2 y 10 mon	I	0	NA			NA			NA			3 y 4 mon	I	1	4 y 9 mon	N	1
6	F	4 mon	6 mon	8 mon	2 y	3 y	1 y 2 mon	5 y	II	0	9 y	II	0	10 y 6 mon	II	0	12 y 6 mon	II	1	14 y 4 mon	III	1	15 y 10 mon	III	1

*F* female, *M* male, *HCt* head control, *RO* roll over, *IS* independent sitting, *IW* independent walking, *WS* walk stably, *WE* word expression, *G* gross motor function classification system, *C* cognitive ability of word expression system, *N* normal, *NA* not available

**Table 3:** Mental developmental milestones and school performance of 6 patients

Pt	Sex	Age*	S	RP	WE	SP
1	M	10 y 9 mon	1 mon	11 mon	1 y 1 mon	Above the average all the time, now at Grade 6, not good at reading comprehension
2	M	9 mon	NA	6 mon	NA	NA
3	M	6 y 1 mon	3 mon	5 mon	1 y 6 mon	Average at kindergarten
4	M	3 y 8 mon	NA	12 mon	NA	Not go to school
5	M	4 y 9 mon	3 mon	7 mon	1 y	Not go to school
6	F	15 y 10 mon	5 mon	5 mon	1 y 2 mon	Above the average at the kindergarten, Grade 1 and 2, below average at Grade 3, dropped out at Grade 6 in primary school because of motor deterioration, not good at mathematics

*F* female, *M* male, Age* age at last follow-up, *S* smile, *RP* recognize people, *WE* word expression, *SP* school performance, *NA* not available

**Fig. 1:**
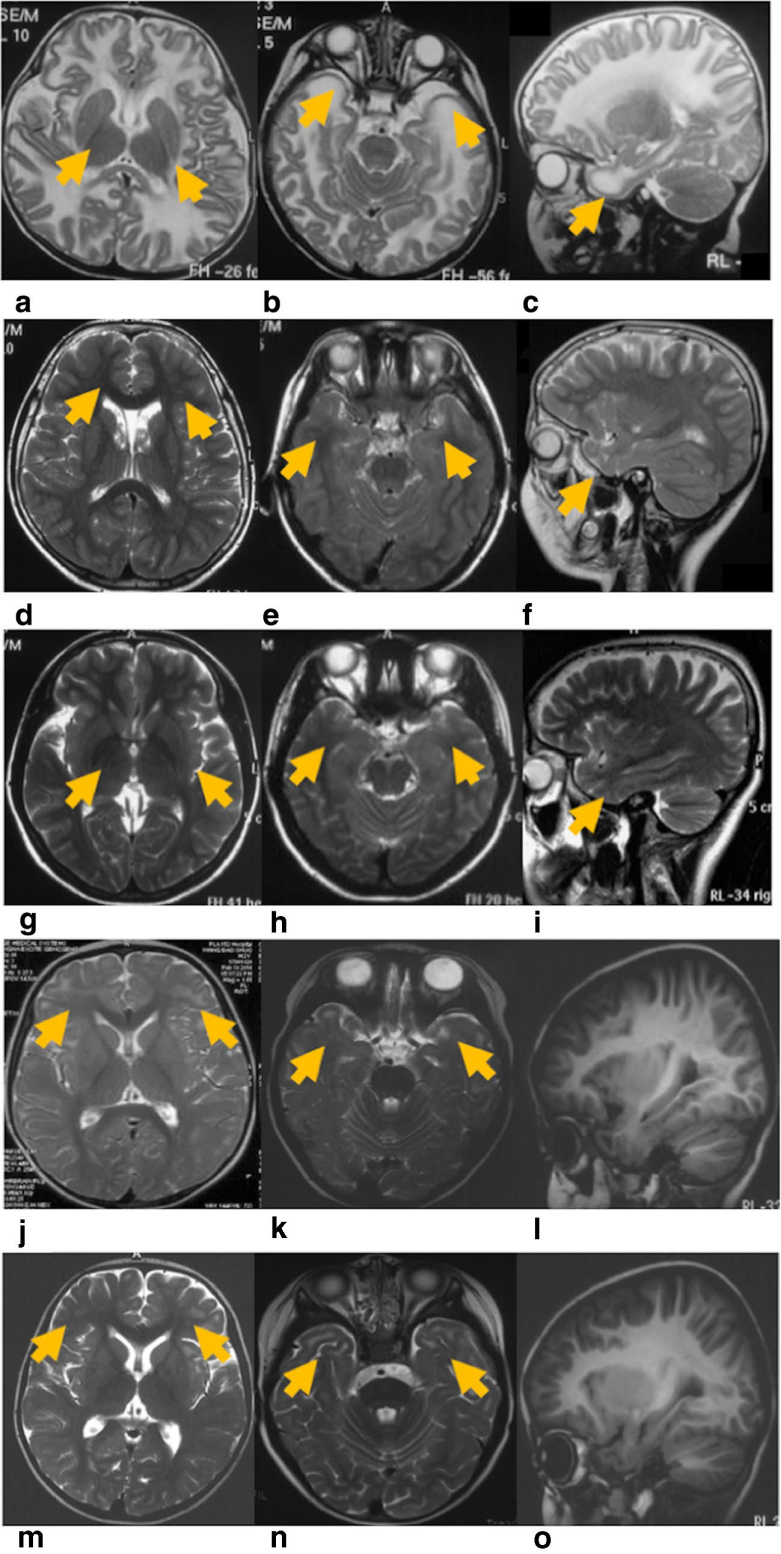
MRI of Pt1 and Pt5. T2WI, T2 weighted-image. **a**–**i** MRI of Pt1. **a**–**c** MRI of Pt1 at 7 m; **a** Bilateral diffuse abnormal signals in subcortical and central white matter with abnormal signals (arrows) in the partial posterior limb of the internal capsule in axial T2WI; **b** temporal cysts were detected in axial T2WI (arrows); **c** temporal cysts in sagittal T2WI (arrows); **d**–**f** MRI of Pt1 at 7 years 9 months. Normal white matter signals without temporal cysts; **g**–**i** MRI of Pt1’s mother at 35 years 1 month, normal signals in white matter without temporal subcortical cysts; **j**–**o** MRI of Pt5. **j**–**l** MRI of Pt5 at 2 years 4 months; **j** bilateral abnormal signals in subcortical white matter (arrows) with normal central white matter and without cysts in axial T2WI; **k**, **l** abnormal signals in subcortical white matter in axial and sagittal T1WI; **m**–**o** MRI of Pt5 at 2 years 6 months. No improvement was seen in cerebral white matter

In the follow-up study, one patient (Pt2) was missing and the remaining five patients attended for 5 (Pt1), 4 (Pt3), 2 (Pt4), 2 (Pt5), and 5 (Pt6) times of follow-up study. HC above the 97th percentile of normal children was found in Pt1, Pt3, Pt4, and Pt6, and a normal HC was found in Pt5 at the first visit (Fig. S1). The HC showed a rapid growth and then slowed down after 2 years in Pt5. GMFCS classification improved in Pt1, Pt3, and Pt5 from GI to normal, stabilized in Pt5 at GI, and declined in Pt6 from GII to GIII (Table 2). The cognitive classification declined in four patients, except Pt1 (Table 2); Pt3 and Pt5–Pt6 declined from normal to C1, whereas Pt4 declined from C1 to C3. School performance was above average for those who attended school, and Pt6 dropped out of school at 11 years because of motor disability. Additionally, language absence and autism only appeared in Pt4. Focal epilepsy occurred four times in 2 months and was controlled by sodium valproate in Pt5. Pt6 had two unconsciousness episodes after head trauma with motor deterioration. The MRI was typical in four patients (Pt1–Pt3 and Pt6), with bilateral diffuse abnormal signals in swollen cerebral WM with or without subcortical cysts, and atypical with only subcortical WM involved in Pt5; the involvement of the posterior limbs of the internal capsule (PLIC) was discovered in the MRI in Pt1 and Pt3, no anterior limbs of internal capsule (ALIC) were found. Thorough MRI improvement was detected in Pt1 (Fig. 1).

#### Genetic findings

Four promising variants in *GLIALCAM* were detected in six patients were all genetically diagnosed (Table 4). One patient (Pt6) presented with compound heterozygous variants c.203A>T (p.Lys68Met) and c.395C>A (p.Thr132Asn) in *GLIALCAM*, while the five remaining patients (Pt1–Pt5) harbored only one variant in *GLIALCAM*. Of the five patients with mono-allelic variant in *GLIALCAM*, four patients (Pt1–Pt3, and Pt5) shared the same variant c.274C>T(p.Arg92Trp), which has been reported in patients with MLC before [4], while Pt4 had a novel variant c.275G>C(p.Arg92Pro) (Fig. S3), which has not been reported before. Except the variant in Pt3 arising de novo, variants in Pt1, Pt2, Pt4, and Pt5 were all inherited from their mothers. The mothers of Pt1 and Pt4 had a history of transient macrocephaly.

**Table 4:** *GlialCAM* variants with pathogenicity analysis

Pt	Variants	Protein	Novel/reported	Origin	GERP	Polyphen2	Mutation taster	SIFT	CADD	M-CAP	Condel	ACMG
1	c.274C>T	p.Arg92Trp	Reported	M*	5.84	PD(0.99)	DC(0.999954)	D(0)	29.2	D(0.88394)	D(0.886)	Pathogenic
2	c.274C>T	p.Arg92Trp	Reported	M	5.84	PD(0.99)	DC(0.999954)	D(0)	29.2	D(0.88394)	D(0.886)	Pathogenic
3	c.274C>T	p.Arg92Trp	Reported	De novo	5.84	PD(0.99)	DC(0.999954)	D(0)	29.2	D(0.88394)	D(0.886)	Pathogenic
4	c.275G>C	p.Arg92Pro	Novel	M*	5.84	PD(0.89865)	DC(1)	D(0)	34	D(0.89357)	D(0.945)	Likely
												Pathogenic
5	c.274C>T	p.Arg92Trp	Reported	M	5.84	PD(0.99)	DC(0.999954)	D(0)	29.2	D(0.88394)	D(0.886)	Pathogenic
6	c.203A>T	p.Lys68Met	Reported	M	5.84	PD(1)	DC(0.999994)	D(0)	28.3	D(0.87393)	D(0.945)	Likely
												Pathogenic
	c.395C>A	p. Thr132Asn	Reported	P	5.97	PD(0.995)	DC(0.999993)	D(0)	25.5	D(0.77129)	D(0.902)	Uncertain
												Significance

*P* paternal, *M* maternal, M* mother with macrocephaly history, *PD* probably deleterious, *DC* disease causing, *D* deleterious

All identified variants in *GLIALCAM* were four missense variants. All changed sites were analyzed to be highly conservative by GERP. All of the four variants were unrecorded in gnomAD database and predicted to be deleterious by Polyphen2, SIFT, Mutation Taster, and Model.

According to the ACMG guideline [18], the variants c.274C>T (p.Arg92Trp), c.275G>C(p.Arg92Pro), c.203A>T (p.Lys68Met), and c.395C>A (p.Thr132Asn) were classified as “pathogenic,” “likely pathogenic,” “likely pathogenic,” and “uncertain significance,” respectively.

### Brain histopathological findings

#### Validation of the GLIALCAM point mutation in mouse models

Genotype verification was performed by PCR and Sanger sequencing at postnatal 7 days for every mouse born from the crossbred of *Glialcam*^Arg92Trp/+^ with C57Bl/6N wild-type mice or *Glialcam*^Lys68Met/+^ and *Glialcam*^Thr132Asn /+^ mice (Fig. S2B).

#### Pathological results

**Fig. 2:**
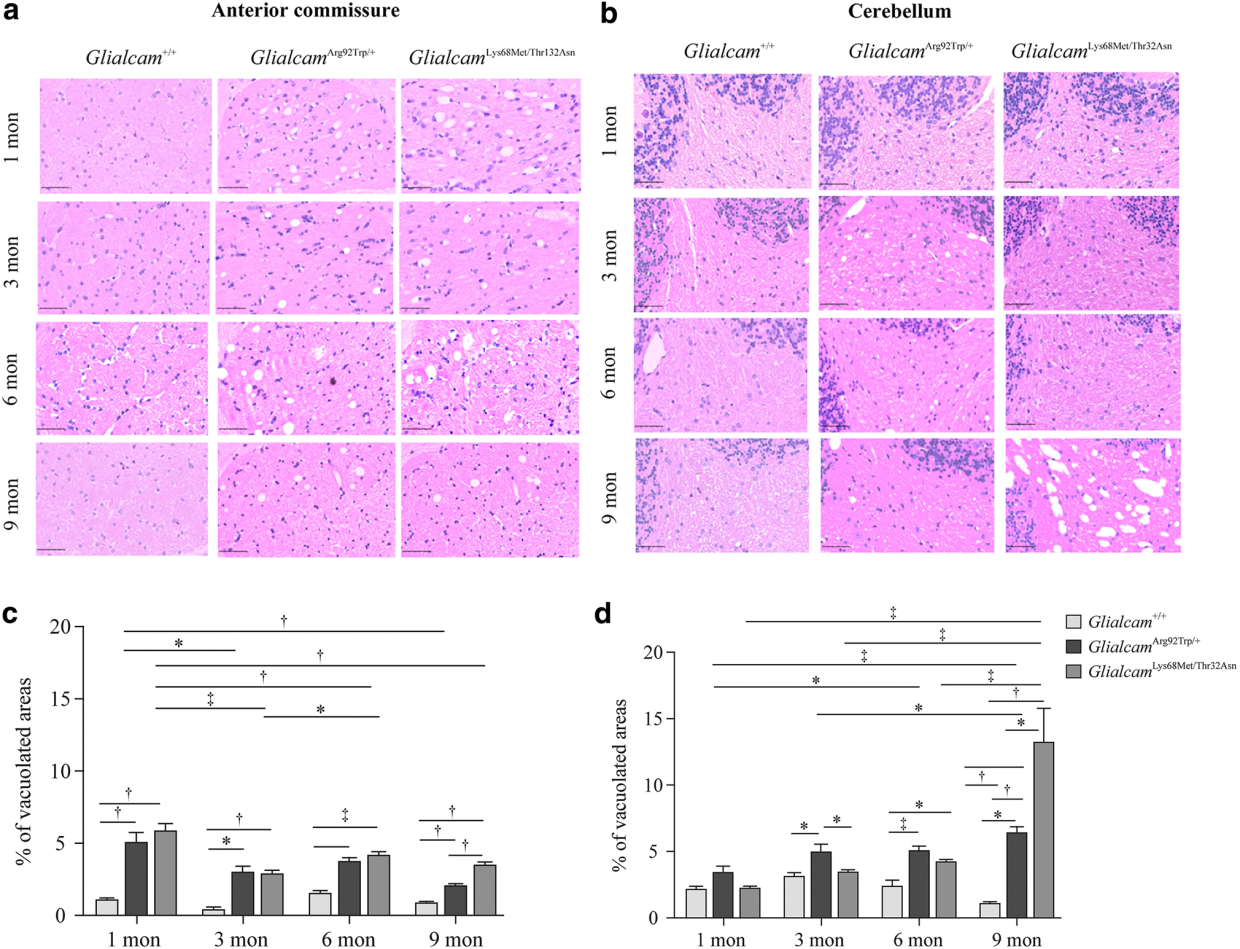
White matter vacuolization in *Glialcam*^Arg92Trp/+^ and *Glialcam*^Lys68Met/Thr132Asn^ mice in the anterior commissural and cerebellar WM. **a**, **b** HE staining of mice aged 1 month, 3 months, 6 months, and 9 months shows vacuolization in white matter. In the anterior commissure, vacuolization starts from 1 month in *Glialcam*^Arg92Trp/+^ and *Glialcam*^Lys68Met/Thr132Asn^ mice, and *Glialcam*^Lys68Met/Thr132Asn^ has more vacuoles than *Glialcam*^Arg92Trp/+^ mice at 9 months. In cerebellum white matter, vacuolization in *Glialcam*^Arg92Trp/+^ mice started from 3 months; in *Glialcam*^Lys68Met/Thr132Asn^ mice, vacuolization started from 6 months, and the degree of vacuolization was more prominent than that in *Glialcam*^Arg92Trp/+^ mice at 9 months. Scale bars 50 µm. **c**, **d** Quantification confirms anterior commissural vacuolization in *Glialcam*^Arg92Trp/+^, *Glialcam*^Lys68Met/Thr132Asn^ has more prominent vacuoles than the wild type with a decreased tendency, and *Glialcam*^Lys68Met/Thr132Asn^ has more vacuolization than *Glialcam*^Arg92Trp/+^ mice at 9 months. **c**, **d** show significantly more prominent white matter cerebellar vacuolization of *Glialcam*^Lys68Met/Thr132Asn^ mice than *Glialcam*^Arg92Trp/+^ mice at 9 months with an increased tendency. *P* < 0.05, †*P* < 0.01 and ‡*P* < 0.001. Graph bars represent the standard error of the mean

**Fig. 3:**
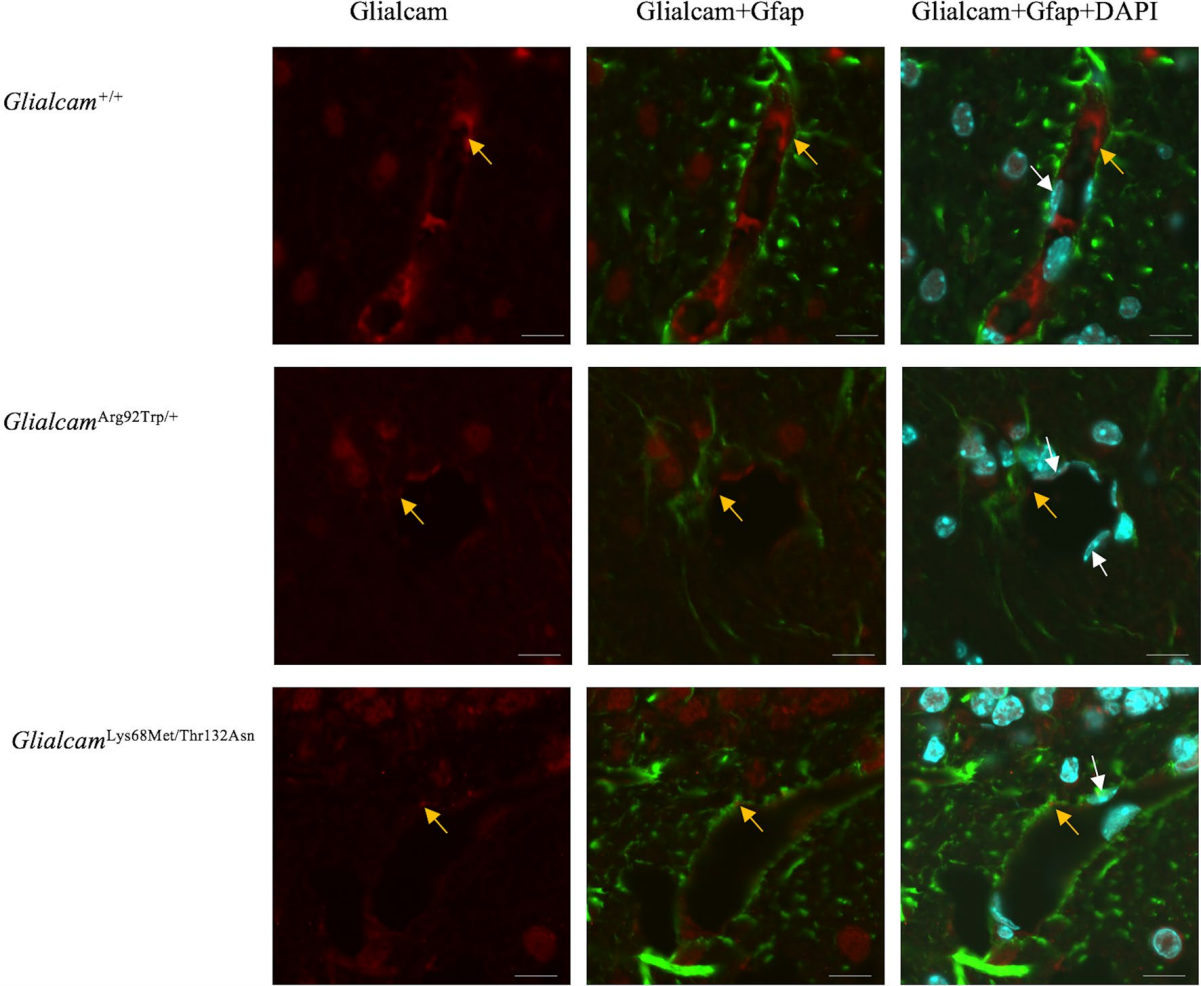
Glialcam expression decreased in astrocyte along blood vessels in cerebellum at 9 months. Fluorescence immunohistochemistry staining of Glialcam (green) and the astrocytic cytoskeletal protein Gfap (red) in the white matter of the cerebellum. Arrows (white) point to nucleus (blue) of endothelial cell, and arrows (yellow) point to the Glialcam protein in astrocyte along blood vessels. It seemed that Glialcam showed a decreased red fluorescent in *Glialcam*^Arg92Trp/+^ and *Glialcam*^Lys68Met/Thr132Asn^ mice brain. Scale bars 20 μm

Vacuoles were detected in the WM of *Glialcam*^Arg92Trp/+^ and *Glialcam*^Lys68Met/Thr132Asn^ mice through brain histopathology. The vacuolization of anterior commissural WM started within 1 month of age in *Glialcam*^Arg92Trp/+^ and *Glialcam*^Lys68Met/Thr132Asn^ models and showed a decreased tendency in subsequent months (Fig. 2a). *Glialcam*^Lys68Met/Thr132Asn^ mice presented a heavier degree of vacuolization than *Glialcam*^Arg92Trp/+^ mice at 9 months in anterior commissure. No vacuoles were detected in the anterior commissural WM of wild-type mice. The vacuoles in *Glialcam*^Arg92Trp/+^ murine cerebellum were detected at 3 months, prominent at 6 months, and increased at 9 months, while the vacuoles in *Glialcam*^Lys68Met/Thr132Asn^ were first detected at 6 months, increased at 9 months (Fig. 2b), and became more prominent than *Glialcam*^Arg92Trp/+^ mice at 9 months. Quantification analysis showed these results (Fig. 2c, d). Glialcam expression and localization of astrocytes along blood vessel in the cerebellum at 9 months were shown (Fig. 3), indicating decreased Glialcam fluorescent in the brains of *Glialcam*^Arg92Trp/+^ and *Glialcam*^Lys68Met/Thr132Asn^ mice comparing with wild-type mice; and no obvious mislocalization of Glialcam and Gfap were found.

## Discussion

In this study, macrocephaly, normal or slightly delayed in motor and cognitive development, and classic MRI features were found in five of the six patients evaluated. Therefore, the five patients were clinically diagnosed. The other patient had HC > 1 SD and a rapidly increased macrocephaly history during the infant period, with only subcortical WM involved; this patient was clinically diagnosed as atypical MLC. Four missense variants c.274C>T (p.Arg92Trp), c.275G>C(p.Arg92Pro), c.203A>T (p.Lys68Met) and c.395C>A (p.Thr132Asn) were detected in the six patients. One patient (Pt6) had two compound heterozygous mutations, and the five other patients had one mono-allelic mutation (Pt1–Pt5). Therefore, the six patients were genetically diagnosed. Five patients (Pt1–Pt5) had MLC2B, and one patient (Pt6) had MLC2A.

Previous studies reported that macrocephaly is the most common presenting symptom in the patients with MLC [1, 4, 8]; in the present study, it appeared in five of the six patients. In the long term, HC growth varied among the patients. Rapid growth after 2 years was found in Pt3 and Pt5 and was first discovered in patients with *GLIALCAM* mutations. The HC of four patients with MLC2B (Pt1–Pt4) were always above 97th of the control at the fifth follow-up study, but macrocephaly was normalized in one of the patient with MLC2B in the follow-up studies. Pt5 presented with normal HC all the time and with a rapid HC growth in the infant period, which was reported previously [4, 8, 12, 17]. The motor development of five patients with MLC2B developed stably or faster, indicating an improvement; meanwhile, that of patient with MLC2A declined because of motor retrogress. Early motor development is normal before walking [4]. The changes in the GMFCS of the patients indicate that motor development was kept stable or improved in patients with MLC2B and deteriorated in the patient with MLC2A. Early cognitive milestones were almost normal, but autism and the absence of language appeared in one patient with MLC2B (Pt4), which was reported previously [8]. In the follow-up study, the cognitive development was almost slightly delayed by word expression, and the school performance was almost normal. Almost all patients with MLC2A and MLC2B were detected with classic MRI, but atypical MRI involving subcortical WM only was found in one patient with MLC2B (Pt5). One patient with MLC2B showed improved MRI; this phenomenon was also reported in literature [8]. A double line throughout involvement was the most common abnormal signal of the internal capsule in patients with MLC1 and MLC2A [1, 8]. The present study is the first to report the involvement of the posterior limb of the internal capsule in the patients (Pt1 and Pt3). Regarding other neurological dysfunction, autistic behavior was frequently found in patients with MLC2B and rare in patients with MLC1; thus far, autistic behavior has not been reported in patients with MLC2A [4, 8, 16]. This finding could be used to distinguish MLC2B in patients with MLC1 and MLC2A clinically. Therefore, combining the clinical and genetic results revealed that the compound heterozygous mutations of *GLIALCAM* caused deteriorated progress, while dominant mutations lead to improved phenotype with macrocephaly and delayed development with or without macrocephaly. Atypical presentations including the involvement of subcortical white matter on MRI, a normalized head circumference on follow-up, or the absence of language and autism appear in patients with MLC2B.

Thus far, 19 mutations have been reported in *GLIALCAM*, including 6 mutations associated with MLC2B and 13 mutations associated with MLC2A. One novel mutation c.275G>C (p.Arg92Pro) was found in the present study, expanding the spectrum of *GLIALCAM* mutations. Interestingly, another substitution at the same nucleotide site [c.275G>A (p.Arg92Gln)] was detected in a patient with MLC2A [4]. The different inheritance patterns and clinical presentation of p.Arg92Pro and p.Arg92Gln, possibly resulting from the replacement by different amino acid residues from basic hydrophilic (Arg) to neutral hydrophobic (Pro) and neutral hydrophilic (Gln), might alter the secondary structure and affect the function of the protein. However, the mechanism needs to be explored further. The mutation c.274C>T (p.Arg92Trp) accounted for four of the five patients with MLC2B. A previous study also detected this mutation, which accounted for 28.5% of the reported patients with MLC2B [4]. Thus, c.274C>T (p.Arg92Trp) was supposed to be a hot-spot mutation.

*Glialcam*-null mice and *Glialcam*^Gly89Ser/+^ mouse models were generated previously [19–21]. *GLIALCAM* mutations with autosomal dominant inheritance result in an improved phenotype, while *GLIALCAM* mutations with autosomal recessive inheritance lead to motor deterioration and mental decline in patients with MLC. *Glialcam* mutation mouse models can be used to investigate brain histopathological differences and prognosis of different inheritances of MLC. Here, we generated the first *Glialcam*^Lys68Met/Thr132Asn^ mouse model with an autosomal recessive inherited manner and a new *Glialcam*^Arg92Trp/+^mouse model with an autosomal dominant inherited manner. No MRI abnormality was observed during the entire period in *Glialcam*^Arg92Trp/+^ and wild-type mice (data not shown). The HE staining on the brain tissue slices was conducted to observe the pathological changes. Vacuoles were detected in the anterior commissural WM in *Glialcam*^Arg92Trp/+^ and *Glialcam*^Lys68Met/Thr132Asn^ mice at 1 month, indicating that the pathological change started from the infant period, consistent with findings in human patients. Thereafter, the vacuoles showed a decreased tendency at subsequent time points, a similar phenomenon observed in the corpus callosum *of Glialcam*-null mice [21]. No vacuoles were found in wild-type mice. In the cerebellar WM, *Glialcam*^Arg92Trp/+^mice showed pathological changes earlier than *Glialcam*^Lys68Met/Thr132Asn^, both with increased tendency but more severe pathological changes appeared in *Glialcam*^Lys68Met/Thr132Asn^ than *Glialcam*^Arg92Trp/+^ at middle age. Thus, the WM vacuoles appeared in both mouse models within the infant period and increased in the cerebellum during the disease course. *Glialcam*^Lys68Met/Thr132Asn^ mice showed more severe vacuolization in the anterior commissure and cerebellum than *Glialcam*^Arg92Trp/+^and wild-type mice at middle ages, indicating the more severe involvement of brain tissues in the former. No improvement occurred in *Glialcam*^Arg92Trp/+^mice at the end of the observation, which are different from the results in some patients with *GLIALCAM*^Arg92Trp/+^ mutation. The difference in the vacuolization timeline in white matter between patients and mice may be due to their different development times or anatomic structures. The maximum age observed is 9 months, and whether the remitting of cerebellar WM vacuolization appears or not remains to be seen. Therefore, the pathology in mice with bi-allelic mutations in *Glialcam* was more severe than that with mono-allelic mutation over time, consistent with the prognosis of human patients. *Glialcam*^Arg92Trp/+^ mice did not show remitting in contrast to some patients with MLC2B and the same mutation. The expression of Glialcam in astrocytes along blood vessels of cerebellar white matter at 9 months were decreased, indicating that the decreased expression of Glialcam may contribute to the vacuolization, but Western Blot are needed to verify this in the future. There was no obvious difference of localization between Glialcam and Gfap in three group.

The clinical and genetic characteristics of patients with MLC and *GLIALCAM* mutations were revealed. One novel mutation was identified, expanding the spectrum of *GLIALCAM* mutations. The first *Glialcam* mouse models with autosomal recessive inheritance and a new *Glialcam* mouse model with autosomal dominant inheritance were generated. The two mouse models with different inheritance patterns showed different degrees of brain pathological features, which were consistent with patients’ phenotype and further confirmed the pathogenicity of the corresponding mutations.

## Electronic supplementary material

Below is the link to the electronic supplementary material.

Supplementary file1 (DOCX 1829 kb)
