# Supplementary material for: Identification in Chinese patients with GLIALCAM mutations of megalencephalic leukoencephalopathy with subcortical cysts and brain pathological study on Glialcam knock-in mouse models
Source: World J Pediatr. 2019 Aug 1;15(5):454–64. doi: 10.1007/s12519-019-00284-w (PMC6785595; doi:10.1007/s12519-019-00284-w)
Supplement: Supplementary file 1 — Supplementary file1 (DOCX 1829 kb) [file 12519_2019_284_MOESM1_ESM.docx]

**Table S1. The sex and generations of the animals used for the experiment**

| ID | WT | | R92W | | K68M+T132N | |
| --- | --- | --- | --- | --- | --- | --- |
|  | generation | sex | generation | sex | generation | sex |
| 1M-1 | F1-1-1 | ♂ | F1-1-1 | ♂ | F1-1-1 | ♂ |
| 1M-2 | F1-1-2 | ♂ | F1-1-2 | ♂ | F1-1-2 | ♂ |
| 1M-3 | F1-1-3 | ♀ | F1-1-3 | ♀ | F1-2-1 | ♂ |
| 3M-1 | F1-2-1 | ♀ | F2-1-1 | ♀ | F1-1-3 | ♀ |
| 3M-2 | F1-2-2 | ♀ | F2-1-2 | ♀ | F1-2-2 | ♀ |
| 3M-3 | F1-2-3 | ♀ | F2-1-3 | ♀ | F1-2-3 | ♀ |
| 6M-1 | F2-1-1 | ♂ | F2-2-1 | ♂ | F2-2-1 | ♂ |
| 6M-2 | F2-1-2 | ♂ | F2-2-2 | ♂ | F2-2-2 | ♂ |
| 6M-3 | F2-1-3 | ♂ | F2-2-3 | ♂ | F2-2-3 | ♂ |
| 9M-1 | F1-3-1 | ♂ | F1-2-1 | ♂ | F2-1-2 | ♂ |
| 9M-2 | F1-3-2 | ♂ | F1-2-2 | ♂ | F2-1-3 | ♂ |
| 9M-3 | F1-3-3 | ♂ | F1-2-3 | ♂ | F2-1-4 | ♂ |

**Abbreviations: M, month; F, ♂, male mice; ♀, female mice**


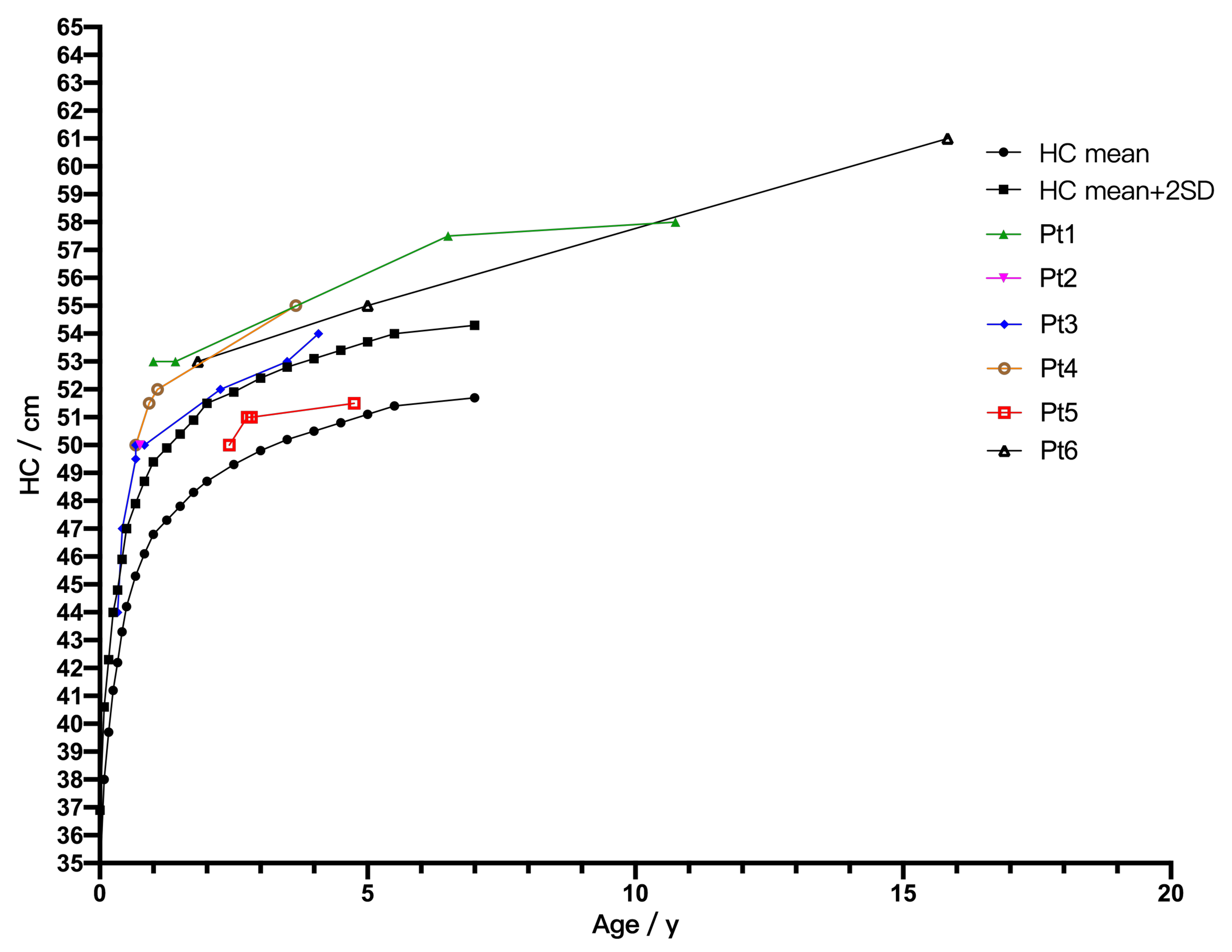


Fig. S1. Head circumference of patients in the follow-up study. y, years. HC of Pt1 and Pt3–Pt6 until the fifth follow-up study.


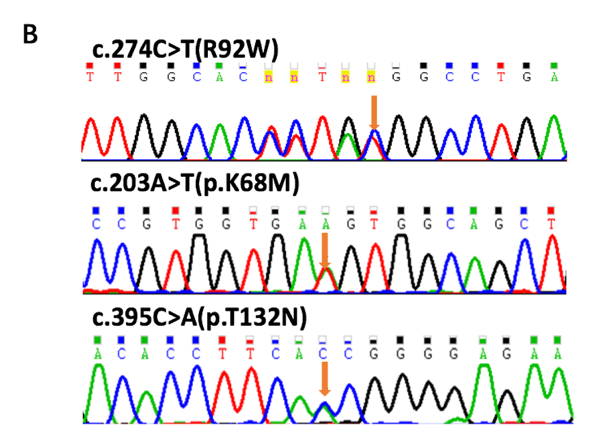

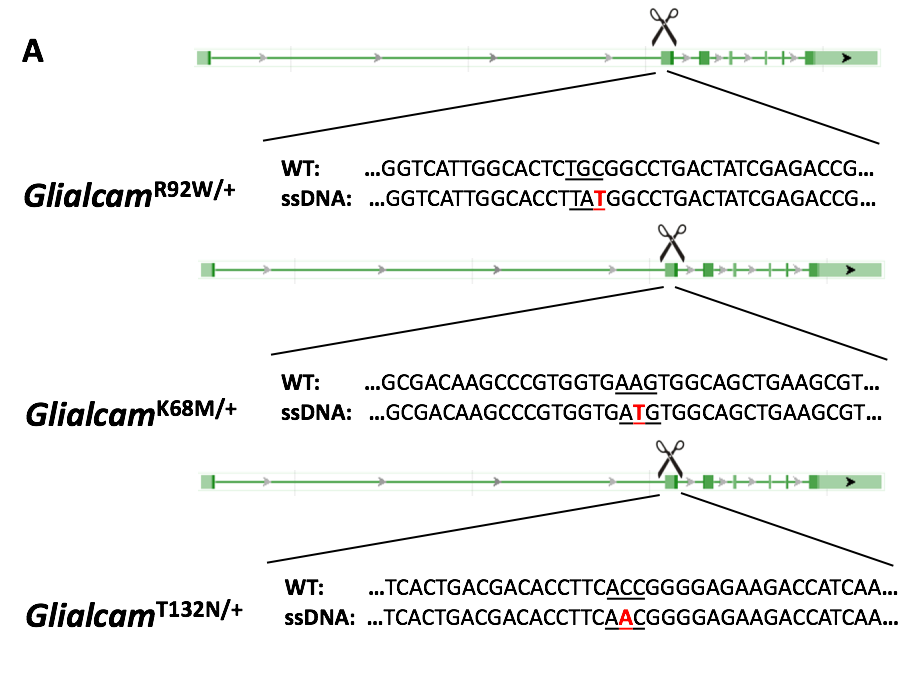


**Fig S2. Generation of the *Glialcam*^R92W/+^****, *Glialcam* ^K68M/+^ and *Glialcam* ^T132N/+^mice.** A. Molecular construction of the *Glialcam*^R92W/+^ ,*Glialcam* ^K68M/+^ and *Glialcam* ^T132N/+^ mice models. B. Sanger sequencing of transgenic mice. Sequencing of *Glialcam*^R92W/+^ mouse (c.274C>T(p.R92W)), and sequencing of *Glialcam* ^K68M/ T132N^ mouse, c.203A>T(p.K68M) and c.395C>A(p.T132N).

**
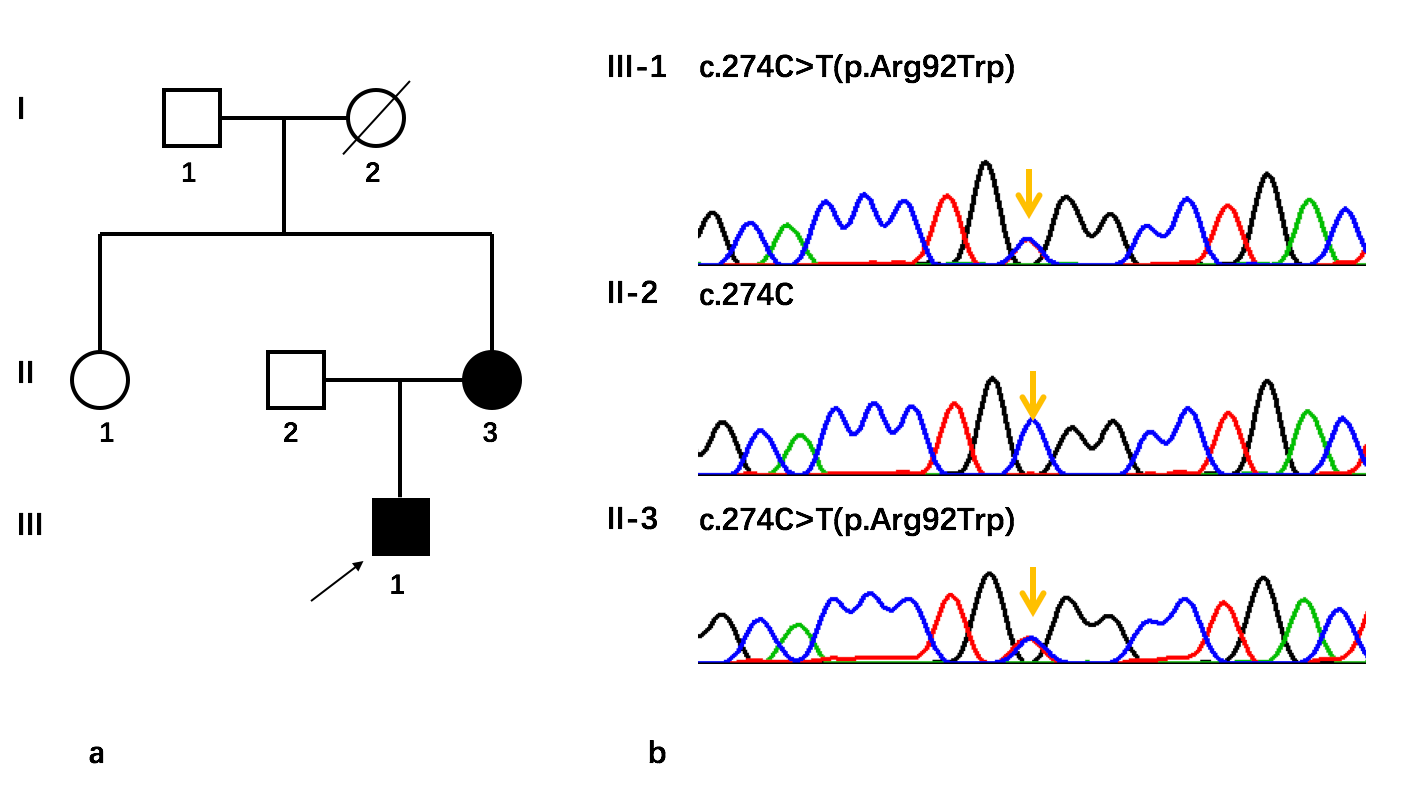
**


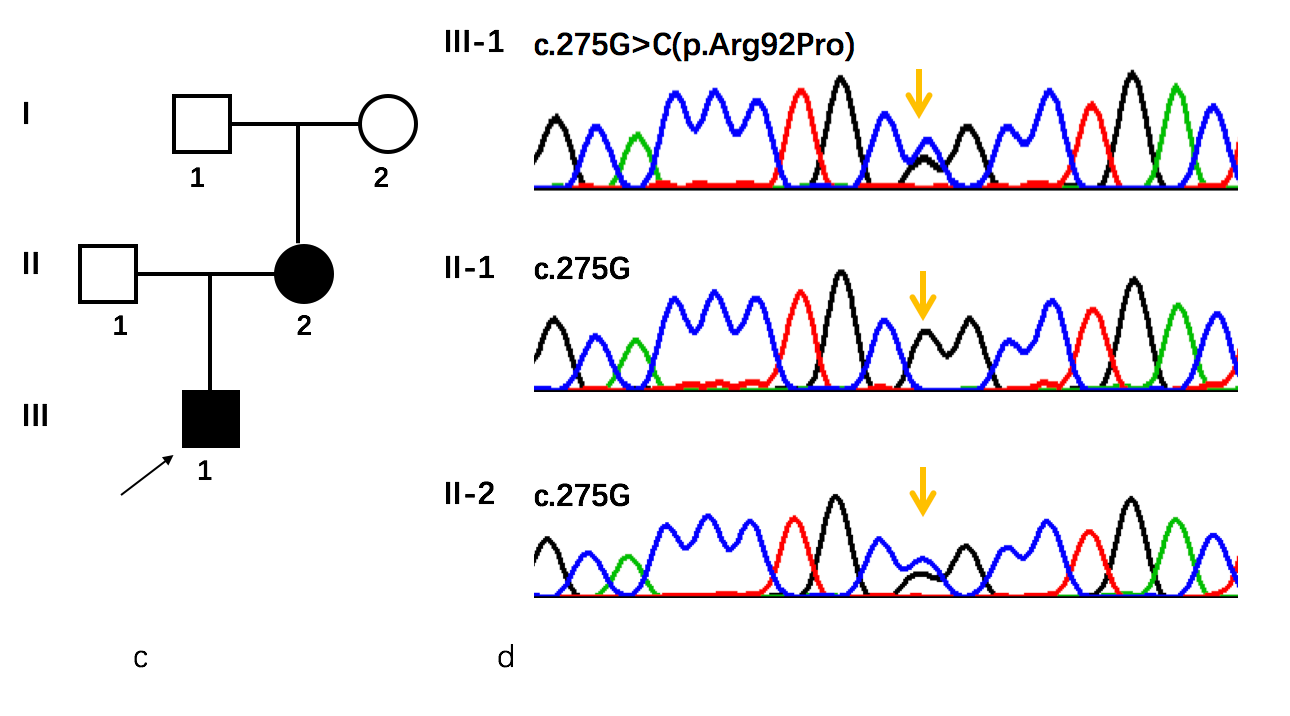


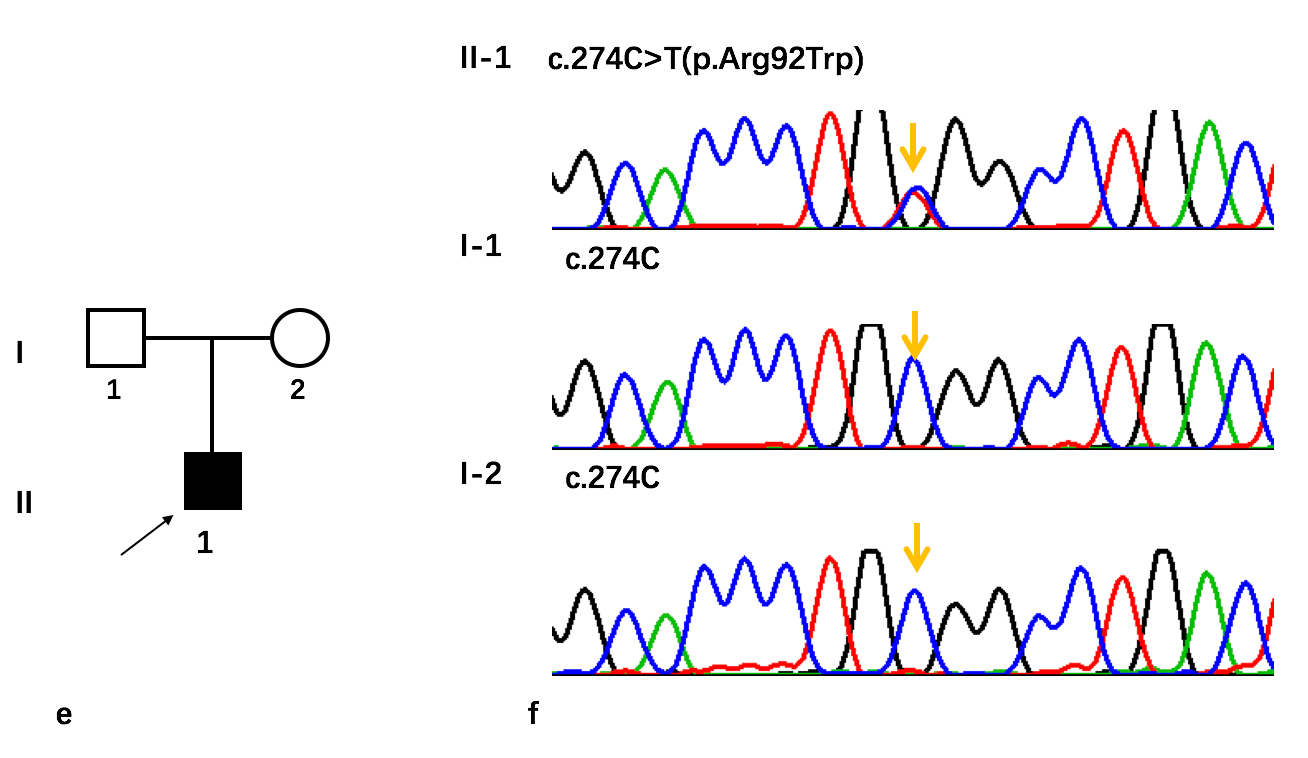


**Fig. S3. Genogram and sequencing results of Pt1, Pt4 and Pt5.** (a) Genogram of Pt1**(III-1)**; (b) c.274C>T(Arg92Tyr) mutation detected in Pt1 and mother; (c) Genogram of Pt4 **(III-1)** ; (d) c.275G>C(p.Arg92Pro)mutation detected in Pt4 and mother. (e) Genogram of Pt5**(II-1)** ; (f) c.274C>T(Arg92Tyr) mutation detected in Pt5.
